# Formula for Migraine

**Published:** 1890-08

**Authors:** 


Formula for Migraine.—Dr. Hammerschlag publishes, in the *Allgemeine medicinische Centralzeitung*, the following prescription, which he has found valuable in migraine:

| | |
|---|---|
| ℞—Caffein citrat | gr. jss. |
| Phenacetin | gr. iij. |
| Sacchar. lacti | gr. v. M. |

Ft. Pulv.

Such a powder may be taken every two hours until the patient is relieved.—*Medical News.*

